# Long-term prognostic value of myocardial work analysis across obesity stages: insights from a community-based study

**DOI:** 10.1038/s41366-025-01863-w

**Published:** 2025-08-06

**Authors:** Fjolla Zhubi Bakija, Máté Tolvaj, Ádám Szijártó, Márton Tokodi, Andrea Ferencz, Bálint Károly Lakatos, Zsuzsanna Ladányi, Loretta Kiss, Zsolt Szelid, Pál Soós, Béla Merkely, Zsolt Bagyura, Attila Kovács, Alexandra Fábián

**Affiliations:** 1 Heart and Vascular Center, Semmelweis University, Budapest, Hungary; 2 Clinic of Cardiology, University and Clinical Center of Kosovo, Prishtina, Kosovo; 3 Department of Experimental Cardiology and Surgical Techniques, Semmelweis University, Budapest, Hungary; 4 Institute for Clinical Data Management, Semmelweis University, Budapest, Hungary

**Keywords:** Cardiovascular diseases, Risk factors

## Abstract

**Background:**

Obesity and overweight are major contributors to cardiovascular disease and adverse outcomes, yet subclinical systolic dysfunction in low-risk individuals often remains undetected by conventional echocardiographic metrics. Myocardial work (MW) analysis offers a more sensitive assessment of left ventricular (LV) function. Thus, we aimed to assess the prognostic value of MW indices in a low-risk, community-based cohort with different stages of obesity.

**Methods:**

We retrospectively identified 1330 volunteers from the Budakalász population-based screening program stratified into 3 groups: patients with normal weight, overweight, and obesity based on BMI. All underwent 2D echocardiography to measure LV ejection fraction (EF), LV global longitudinal strain (GLS), global MW index (GWI), global wasted work (GWW), and global MW efficiency (GWE). The primary endpoint was all-cause mortality over a median follow-up of 11 years.

**Results:**

During follow-up, 138 (10.4%) participants died. LVEF was not predictive of outcome. By univariable Cox regression analysis, GWI was a predictor of outcomes, alongside GWW, GWE, and GLS in the total cohort. In normal weight group, only GLS was a predictor. In the group with overweight, GLS, GWE (HR 0.917 [95%CI 0.874–0.963], *p* < 0.00) and GWW (HR 1.341 [95%CI 1.121–1.604], *p* = 0.001) were predictors of mortality. Among patients with obesity, GWI was the only significant predictor (HR 0.929 [95%CI 0.875–0.986], *p* = 0.015). In patients with overweight and obesity with GWI values below the standard cut-off of 1292 mmHg%, the risk of all-cause mortality was more than 2 times higher.

**Conclusions:**

Myocardial work metrics were significant predictors of long-term outcomes in low-risk individuals with different stages of obesity. Our findings highlight that conventional echocardiographic metrics may underestimate cardiovascular risk in patients with overweight and obesity.

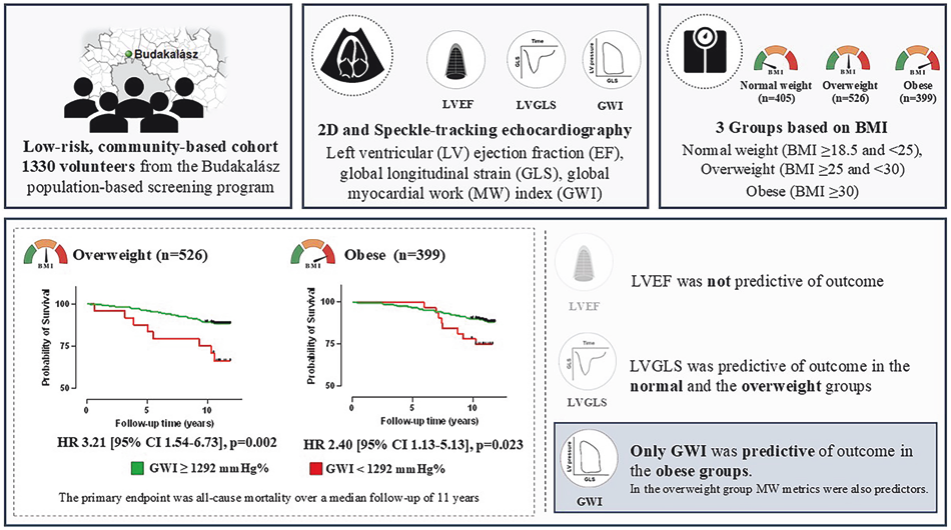

## Introduction

The prevalence of overweight and obesity is rising globally, affecting both developed and developing nations representing a critical and growing health concern. According to the World Health Organization (WHO), ~2.5 billion adults worldwide, around 43% of the global population, are classified as overweight, with a staggering 890 million individuals living with obesity [1]. In European countries affiliated with the European Society of Cardiology, obesity affects about one in five adults [2]. Since 1990, the incidence of adult obesity has more than doubled, contributing to at least 2.8 million deaths annually due to associated health complications [3].

Overweight and obesity are characterized by abnormal or excessive fat accumulation that poses significant health risks [1]. Research has long established a link between obesity and increased mortality, as well as heightened risk for cardiovascular morbidity and mortality [4–7]. While earlier studies primarily attributed the connection between increased adiposity and cardiovascular disease mortality to indirect mechanisms, such as the exacerbation of risk factors and chronic conditions, recent findings indicate that direct mechanisms also contribute to this relationship [8, 9]. Recent studies have shown that individuals previously classified with “metabolically healthy obesity” are at a higher risk of coronary heart disease, cerebrovascular disease, and heart failure compared to their metabolically healthy counterparts of normal weight [8, 10]. This combination of hemodynamic and metabolic stress contributes to elevated cardiovascular risk in individuals with obesity, independent of traditional risk factors.

During the past decades, echocardiography has become a cornerstone screening and diagnostic tool for cardiovascular risk assessment, particularly valuable in low-risk populations due to its non-invasive nature, cost-effectiveness, and accessibility [11–13]. Although left ventricular (LV) ejection fraction (LVEF) remains a primetime measure of systolic function [14, 15], more recent techniques, such as speckle-tracking echocardiography (STE), offer a solution detecting more subtle functional changes. It allows the identification of subclinical LV systolic dysfunction in patients with obesity, indicating systolic dysfunction despite preserved LVEF [16, 17]. Furthermore, noninvasive LV pressure-strain loop-derived myocardial work (MW) provides insights into LV contractility that extend beyond more traditional measures like LVEF and global longitudinal strain (GLS) [18, 19] as it accounts for afterload allowing a more accurate assessment of systolic function [19]. While the relevance of global myocardial work index (GWI) in various cardiac conditions is well-researched, its predictive value in low-risk populations with different stages of obesity still remains understudied [20–22].

Accordingly, our objective was to evaluate the impact of overweight and obesity on MW measures and to assess their prognostic power in a low-risk, community-based cohort.

## Methods

### The Budakalász study cohort

The Budakalász Study was a cross-sectional, voluntary screening initiative targeting the adult population in the Central Hungarian region, designed to collect comprehensive data on their health status and cardiovascular risk profiles and to identify novel cardiovascular risk factors [23]. The inclusion criteria for our current sub-study required the availability of transthoracic echocardiography. The exclusion criteria were absence of apical four-chamber view loops, poor visualization of more than one LV segment in the apical four-chamber view, or suboptimal tracking quality as determined by an expert reader.

### General medical examination

A comprehensive medical history was obtained, focusing on cardiovascular disease assessment and lifestyle factors such as smoking status. The evaluation also included details of current medications. Blood samples were collected for laboratory analysis. Anthropometric data, including weight and height were measured to calculate body mass index (BMI) and body surface area (BSA) using validated equipment. A 12-lead electrocardiogram (ECG) was conducted [23]. Blood pressure readings were taken on both arms with the participant in a supine position following a 20-min rest after the echocardiographic examination. Hypertension, hyperlipidemia, or diabetes mellitus were considered present if previously diagnosed or under treatment, as recorded in the medical history.

### Echocardiographic assessment

Echocardiographic image acquisitions were performed using a commercially available ultrasound system (Vivid i, 3Sc-RS transducer) by three experienced echocardiographers. All participants underwent a standardized image analysis protocol, which included two-dimensional (2D) echocardiography, Tissue Doppler imaging (TDI) and STE. The acquired echocardiographic images were analyzed by two experienced readers, both of whom were blinded to the clinical outcomes, using the commercially available software for offline analysis (Ultrasound Workspace, Philips Medical Systems, The Netherlands).

### Conventional echocardiography

LV internal diameters, wall thicknesses, and relative wall thickness were measured. LV mass index (LVMi) was calculated from end-diastolic dimensions using an anatomically validated formula, in accordance with the guidelines of the American Society of Echocardiography [24]. Left atrial end-systolic volume indexed to BSA (LAVi) was measured using the Simpson’s method in the apical four-chamber view [24]. Mitral inflow velocities were assessed using pulsed-wave (PW) Doppler at the level of the mitral leaflet tips in the apical four-chamber view, where peak early (E) and late (A) diastolic inflow velocities were measured to calculate the E/A ratio. Additionally, the deceleration time (DT) of the E-wave was estimated. TDI measurements provided systolic (s′), early diastolic (e′), and late diastolic (a′) velocities of the mitral lateral and septal annulus, and the E/e′ ratio was calculated by dividing the peak trans-mitral E velocity by the averaged e′ velocity from these sites. For right heart assessment, right ventricular basal short-axis diameter (RVd) and tricuspid annular plane systolic excursion (TAPSE) were measured. Right atrial end-systolic volume indexed to BSA (RAVi) was also measured using the Simpson’s method in the apical four-chamber view.

### Speckle-tracking echocardiography

We utilized a commercially available, validated, vendor-independent speckle-tracking software package (AutoStrain LV, Philips Ultrasound Workspace, Philips Medical Systems, The Netherlands) to quantify LVGLS and LV end-diastolic volume, end-systolic volume and LVEF. To minimize patient dropout associated with suboptimal image quality, apical two-chamber and three-chamber views were not included in the analysis. Therefore, volumetric indices and LVEF were measured on apical four-chamber views and LVGLS was calculated by averaging the segmental peak negative strain values of the six LV segments [25]. In instances of suboptimal ECG or 2D echocardiographic image quality or low tracking fidelity, manual corrections to cardiac cycle events or the endocardial contours were performed as necessary.

### Myocardial work analysis

The LVGLS segmental curves were subsequently exported as text files for further analysis using a custom-developed software dedicated to quantifying MW metrics.

When performing MW analysis, we followed the principles previously established by Russell et al. [19, 26]. First, the peak systolic blood pressure values were obtained by performing and averaging brachial artery cuff pressure measurements on both arms. It was considered equivalent to the peak systolic pressure of the left ventricle and was assumed to be uniformly distributed across the ventricle. Then, our custom-developed software automatically reconstructed LV pressure curve by adjusting the previously published LV reference curve according to the non-invasively obtained peak arterial pressure [19]. To determine the timing of valvular events, PW TDI recordings of the mitral lateral annulus were used. To approximate the opening and closure of the aortic and mitral valves, the stages of the cardiac cycle were measured on the recordings as isovolumic contraction time, ejection time, isovolumic relaxation time and filling time. Utilizing these temporal reference points, we proportionally segmented the strain curves into four sections, each corresponding to a phase of the cardiac cycle, and each section of the given strain curve was matched with the corresponding section of the estimated pressure curve. Lastly, the four sections of the recordings were concatenated, and then, using our dedicated software, pressure–strain loops were plotted to enable MW analysis.

MW was quantified by calculating the segmental shortening rate, derived from the differentiation of the strain curve, and multiplying this value by the instantaneous LV pressure. GWI was computed by integrating the power over time from mitral valve closure to mitral valve opening. Global constructive myocardial work (GCW) represents the work generated by the shortening during systole and lengthening during isovolumetric relaxation, whereas global wasted myocardial work (GWW) quantifies the work resulting from lengthening during systole and shortening during the isovolumetric relaxation phase. Finally, global myocardial work efficiency (GWE) was calculated as GCW/(GCW + GWW).

### Study outcomes

Follow-up information (date of death) was obtained from Hungary’s National Health Insurance Database. The primary endpoint of our study was all-cause mortality.

### Statistical analysis

Statistical analysis was performed using dedicated software (SPSS v22, IBM, Armonk, NY, USA). Continuous variables are expressed as mean ± standard deviation, whereas categorical variables were reported as frequencies and percentages. After assessing the distribution of variables using the Shapiro–Wilk test, the clinical and echocardiographic characteristics were compared with unpaired Student’s *t* test or Mann–Whitney U test for continuous variables and chi-squared or Fisher’s exact test for categorical variables, as appropriate. Univariable and multivariable Cox proportional hazard models were used to calculate hazard ratios with 95% confidence intervals (95% CIs). Covariates included in multivariable models were selected based on clinical relevance and intergroup differences. Collinearity of variables was tested at each multivariable model by variance inflation factor (considered collinear if variance inflation factor >3). The previously established lower limits of normal value (GWI value of 1292 mmHg%) [27] were used to dichotomize the study population. Outcomes of the dichotomized groups were visualized on Kaplan–Meier curves and compared by log-rank test. The prognostic performance of the established GWI cutoff was further evaluated using multivariable Cox proportional hazard models, including covariates of clinical relevance and intergroup differences, similarly as described above. A two-sided *P*-value of 0.05 was considered statistically significant.

## Results

### Baseline demographic and clinical characteristics according to primary outcome

A total of 1330 individuals with 2D transthoracic echocardiographic examinations were retrospectively identified. Over a median follow-up period of 11 years, 138 participants reached the primary endpoint of all-cause mortality.

Baseline demographic and clinical characteristics are outlined in Table 1. Individuals with adverse outcomes were significantly older, and there was a significant male predominance in the deceased group (57.2%). Subjects with adverse events had significantly higher BMI values, while BSA values were comparable across groups. Higher systolic and lower diastolic blood pressure was observed among those with adverse outcomes, with no significant difference in heart rate. Regarding cardiovascular risk factors, the most common was hypertension, affecting 47.0% (625 individuals) of the participants, followed by a history of smoking (42%) and diabetes (13%). In terms of laboratory findings, there were no differences between the groups (Table 1).

**Table 1:** Demographic and clinical characteristics of the study samples according to primary outcome.

	Overall (*n* = 1330)	Alive (*n* = 1192)	Deceased (*n* = 138)	*p* value
Baseline demographic characteristics				
Age (years)	53.41 ± 14.70	51.90 ± 13.87	66.43 ± 15.28	**<0.001**
Female, *n* (%)	772 (58.05)	713 (59.82)	59 (42.75)	**<0.001**
Clinical characteristics				
BSA, m^2^	1.90 ± 0.23	1.90 ± 0.23	1.91 ± 0.23	0.893
BMI, kg/m^2^	27.79 ± 5.01	27.65 ± 5.02	29.03 ± 4.78	**0.002**
Systolic blood pressure, mmHg	132.84 ± 18.32	132.00 ± 17.81	140.08 ± 20.95	**<0.001**
Diastolic blood pressure, mmHg	79.40 ± 9.65	79.62 ± 9.57	77.52 ± 10.16	**0.016**
Heart rate, bpm	68.06 ± 9.85	67.96 ± 9.89	68.91 ± 9.49	0.286
Risk factors and medical history				
Smoking history, *n* (%)	557(41.88)	494 (41.44)	63 (45.65)	0.343
Hypertension, *n* (%)	625 (46.99)	560 (46.98)	65 (47.10)	0.978
Diabetes, *n* (%)	169 (12.71)	158 (13.26)	11 (7.97)	0.078
Arrhythmia *n* (%)	138 (10.38)	122 (10.23)	36 (18.85)	0.620
Previous MI, *n* (%)	39 (2.93)	38 (3.19)	1 (0.72)	0.104
Previous PCI, *n* (%)	24 (1.80)	23 (1.93)	1 (0.72)	0.314
Previous CABG, *n* (%)	12 (0.90)	11 (0.92)	1 (0.72)	0.054
Chronic heart failure, *n* (%)	83 (6.24)	81 (6.80)	2 (1.45)	**0.014**
Previous stroke, *n* (%)	62 (4.66)	58 (4.87)	4 (2.90)	0.299
Pulmonary disease, *n* (%)	97 (7.29)	84 (7.05)	13 (9.42)	0.310
Laboratory work				
Total cholesterol, mmol/L	5.48 ± 1.13	5.46 ± 1.14	5.54 ± 1.05	0.523
HDL cholesterol, mmol/L	1.47 ± 0.44	1.46 ± 0.44	1.52 ± 0.42	0.156
LDL cholesterol, mmol/L	3.37 ± 0.97	3.37 ± 0.98	3.41 ± 0.90	0.674
Triglycerides, mmol/L	2.27 ± 1.61	2.28 ± 1.62	2.13 ± 1.49	0.296
Glucose, mmol/L	5.98 ± 1.52	6.00 ± 1.56	5.77 ± 1.08	0.087
ProBNP, pmol/L	126.19 ± 319.66	130.38 ± 335.82	90.13 ± 97.83	0.165
Serum Creatinine, µmol/L	77.29 ± 18.58	77.40 ± 18.69	76.28 ± 17.63	0.502
HBa1c, %	5.74 ± 0.73	5.76 ± 0.75	5.63 ± 0.51	0.050

Continuous variables are presented as means ± SD, categorical variables are reported as frequencies (%). Bold: significant *p* values.

*BMI* body mass index, *BSA* body surface area, *CABG* coronary artery bypass grafting, *HDL* high density lipoprotein, *LDL* low density lipoprotein, *MI* myocardial infarction, *PCI* percutaneous coronary intervention.

### Conventional 2D and speckle-tracking echocardiography-derived parameters according to primary outcome

The conventional 2D echocardiographic parameters are presented in Table 2. Participants with adverse outcomes were presented with marked morphological LV remodeling, namely greater LV internal diameter and higher values of LVMi, LV EDVi, and LV ESVi. Interestingly, LVEF did not differ between groups. In terms of diastolic function, patients with adverse outcomes had significantly higher mitral A-wave velocity and lower E-wave velocity, along with a lower E/A ratio and prolonged DT. Mitral annular early diastolic velocities were lower in the adverse outcome group, and the average E/e′ ratio was higher. Additionally, LAVi was higher in those who met the endpoint. Regarding the right heart, only RAVi was significantly higher in the deceased group, whereas RVd and TAPSE did not differ (Table 2).

**Table 2:** Echocardiographic parameters of study population according to primary outcome.

	Overall (*n* = 1330)	Alive (*n* = 1192)	Deceased (*n* = 138)	*p* value
2D conventional echocardiographic data				
LVIDd, mm	48.29 ± 4.79	48.14 ± 4.72	49.71 ± 5.22	**<0.001**
IVSd, mm	9.94 ± 1.75	9.84 ± 1.67	10.88 ± 2.13	**<0.001**
PWd, mm	9.37 ± 1.51	9.31 ± 1.46	9.91 ± 1.81	**<0.001**
RWT, %	0.44 ± 0.13	0.44 ± 0.13	0.46 ± 0.16	**0.030**
LVMi, g/m^2^	87.50 ± 21.52	85.87 ± 20.04	102.42 ± 28.06	**<0.001**
LV ESVi, ml/m^2^	20.63 ± 4.98	20.46 ± 4.89	22.19 ± 5.52	**<0.001**
LV EDVi, ml/m^2^	59.15 ± 11.09	58.80 ± 11.08	62.25 ± 10.73	**<0.001**
LVEF, %	65.11 ± 5.21	65.19 ± 5.12	64.33 ± 5.92	0.065
*E*, cm/s	76.40 ± 17.69	76.74 ± 16.88	73.36 ± 23.51	**0.037**
*A*, cm/s	68.88 ± 21.00	67.58 ± 20.36	80.28 ± 23.11	**<0.001**
E/A	1.22 ± 0.51	1.24 ± 0.50	1.02 ± 0.58	**<0.001**
DT (ms)	201.89 ± 58.58	198.51 ± 55.87	231.61 ± 72.23	**<0.001**
Mitral lateral s’, cm/s	9.88 ± 2.69	9.95 ± 2.66	9.26 ± 2.87	**0.004**
Mitral lateral e’, cm/s	12.31 ± 4.17	12.58 ± 4.10	10.00 ± 4.01	**<0.001**
Mitral lateral a’, cm/s	10.39 ± 3.18	10.28 ± 3.10	11.30 ± 3.67	**<0.001**
Mitral medial s’, cm/s	8.58 ± 1.79	8.64 ± 1.75	8.08 ± 2.01	**<0.001**
Mitral medial e’, cm/s	9.80 ± 3.38	10.02 ± 3.34	7.88 ± 3.06	**<0.001**
Mitral medial a’, cm/s	10.46 ± 2.42	10.46 ± 2.37	10.53 ± 2.79	0.725
E/e’ average	7.47 ± 2.59	7.28 ± 2.32	9.12 ± 3.95	**<0.001**
LAVi, ml/m^2^	28.94 ± 9.88	28.39 ± 9.42	33.65 ± 12.27	**<0.001**
RVd, mm	34.62 ± 5.41	34.53 ± 5.36	35.40 ± 5.75	**0.073**
RAVi, ml/m^2^	23.13 ± 7.88	22.91 ± 7.80	25.08 ± 8.35	**0.003**
TAPSE, mm	23.85 ± 4.04	23.82 ± 3.99	24.10 ± 4.40	0.457
Speckle tracking echocardiography data				
LVGLS, %	−19.94 ± 3.49	−20.06 ± 3.44	−18.85 ± 3.79	**<0.001**
Myocardial work data				
GWI, mmHg%	2040.65 ± 483.64	2051.02 ± 473.57	1951.05 ± 557.20	**0.021**
GCW, mmHg%	2194.21 ± 472.40	2198.89 ± 463.23	2153.85 ± 545.48	0.289
GWW, mmHg%	144.97 ± 104.14	142.48 ± 102.50	166.51 ± 115.55	**0.010**
GWE, %	93.72 ± 4.27	93.84 ± 4.18	92.68 ± 4.87	**0.003**

Continuous variables are presented as means ± SD. Bold: significant *p* values.

*A* atrial contraction, *a’* peak late (atrial) diastolic annular velocity, *DT* deceleration time, *E* early diastolic filling, *e’* early diastolic annular velocity, *EDVi* end diastolic volume index, *EF* ejection fraction, *ESVi* end-systolic volume index, *GCW* global constructive work, *GLS* global longitudinal strain, *GWE* global work efficiency, *GWI* global work index, *GWW* global wasted work, *IVSd* inter-ventricular septal diameter, *LAVi* left atrial volume index, *LV* left ventricle, *LVIDd* left ventricular internal diameter at end-diastole, *LVMi* left ventricular mass index, *PWd* posterior wall diameter, *RVd* right ventricle diameter, *RWT* relative wall thickness, *s’* systolic annular velocity, *TAPSE* tricuspid annular plane systolic excursion.

STE-derived metrics differed significantly among those with and without adverse outcomes, as shown in Table 2. Subjects with adverse outcomes demonstrated lower LVGLS compared to those without. Regarding MW metrics, GWI values were significantly lower among the deceased group. Moreover, deceased individuals demonstrated higher values of GWW and lower GWE, while GCW did not differ (Table 2).

### Baseline demographic and clinical characteristics according to body mass index

Based on BMI, the total cohort was divided into 3 weight groups. The group with normal weight (*n* = 405) was defined as BMI ≥ 18.5 and <25, the group with overweight (*n* = 526) was defined as BMI ≥25 and <30, whereas the group with obesity (*n* = 399) consisted of patients with BMI ≥ 30 as per WHO data [1]. Table 3 summarizes the demographic and clinical characteristics of each weight group. Participants in the group with normal weight were younger and had higher proportion of females. Compared to the groups with overweight and obesity, the group with normal weight also exhibited significantly lower systolic and diastolic blood pressure. The all-cause mortality rate was higher among individuals with overweight and obesity compared to those in the normal weight group, and interestingly, it was comparable between the groups with overweight and obesity. Regarding cardiovascular risk factors, no significant differences were observed in terms of smoking status, hypertension, or diabetes. Additionally, laboratory parameters did not differ between groups (Table 3). Patients who met the endpoint vs those who did not, were also compared within the three weight groups (Supplementary Table 1). Interestingly, deceased patients among the groups with normal weight and overweight had higher systolic blood pressures, whereas within the group with obesity, there was no difference between the alive and deceased subjects. Moreover, alive and deceased groups did not differ in terms of cardiovascular risk factors (Supplementary Table 1).

**Table 3:** Demographic and clinical characteristics of the study samples according to different weight groups.

	Overall	Normal weight (*n* = 405)	Overweight (*n* = 526)	Obesity (*n* = 399)	*p* value
Baseline demographic characteristics					
Age (years)	53.41 ± 14.70	46.7 ± 15.0^bc^	54.7 ± 14.5^ac^	58.5 ± 11.8^ab^	**<0.001**
Female, *n* (%)	772 (58.05)	290 (71.60)^bc^	240 (45.63)^ac^	242 (39.35)^ab^	**<0.001**
Deceased, *n* (%)	138 (10.37)	37 (5.43)^bc^	67 (12.74)^a^	49 (12.28)^a^	**<0.001**
BSA, m^2^	1.90 ± 0.23	1.72 ± 0.17^bc^	1.92 ± 0.18^ac^	2.06 ± 0.19^ab^	**<0.001**
BMI, kg/m^2^	27.79 ± 5.01	22.36 ± 1.90^bc^	27.40 ± 1.39^ac^	33.81 ± 3.35^ab^	**<0.001**
Systolic blood pressure, mmHg	132.84 ± 18.32	126.25 ± 19.34^bc^	134.19 ± 17.76^ac^	137.75 ± 15.94^ab^	**<0.001**
Diastolic blood pressure, mmHg	79.40 ± 9.65	77.13 ± 10.06^bc^	79.24 ± 9.07^ac^	81.92 ± 9.38^ab^	**<0.001**
Heart rate, bpm	68.06 ± 9.85	66.81 ± 9.78^c^	67.57 ± 9.83^c^	69.92 ± 9.70^ab^	**<0.001**
Risk factors and medical history					
Smoking history, *n* (%)	557(41.88)	161 (39.85)	213 (40.49)	183 (48.86)	0.152
Hypertension, *n* (%)	625 (46.99)	177 (43.81)	256 (48.67)	192 (48.12)	0.279
Diabetes, *n* (%)	169 (12.71)	47 (11.63)	66 (12.55)	56 (14.04)	0.580
Arrhythmia *n* (%)	138 (10.38)	45 (11.14)	50 (9.51)	43 (10.78)	0.693
Previous MI, *n* (%)	39 (2.93)	10 (2.48)	9 (1.71)	20 (5.01)	**0.010**
Previous PCI, *n* (%)	24 (1.80)	6 (1.49)	12 (2.28)	6 (1.50)	0.572
Previous CABG, *n* (%)	12 (0.90)	6 (1.49)	4 (0.76)	2 (0.50)	0.308
Chronic heart failure, *n* (%)	83 (6.24)	29 (7.18)	33 (6.27)	21 (5.26)	0.538
Previous stroke, *n* (%)	62 (4.66)	15 (3.71)	32 (6.08)	15 (3.76)	0.138
Pulmonary disease, *n* (%)	97 (7.29)	23 (5.69)	41 (7.79)	33 (8.27)	0.313
Laboratory work					
Total cholesterol, mmol/L	5.49 ± 1.14	5.56 ± 1.13	5.48 ± 1.15	5.41 ± 1.09	0.186
HDL cholesterol, mmol/L	1.48 ± 0.45	1.45 ± 0.42	1.49 ± 0.46	1.45 ± 0.41	0.275
LDL cholesterol, mmol/L	3.37 ± 0.97	3.45 ± 0.95	3.36 ± 0.99	3.30 ± 0.96	0.099
Triglycerides, mmol/L	2.27 ± 1.65	2.30 ± 1.61	2.25 ± 1.71	2.26 ± 1.47	0.877
Glucose, mmol/L	5.96 ± 1.51	6.04 ± 1.66	5.92 ± 1.35	6.00 ± 1.58	0.732
ProBNP, pmol/L	133.13 ± 421.99	132.23 ± 436.91	118.44 ± 206.93	130.22 ± 301.04	0.776
Serum Creatinine, µmol/L	77.15 ± 18.72	77.71 ± 16.72	77.12 ± 20.72	77.08 ± 17.39	0.860
HBa1c, %	5.73 ± 0.72	5.76 ± 0.75	5.75 ± 0.72	5.71 ± 0.72	0.572

Continuous variables are presented as means ± SD, categorical variables are reported as frequencies (%). Bold: significant *p* values.

*BMI* body mass index, *BSA* body surface area, *CABG* coronary artery bypass grafting, *HDL* high density lipoprotein, *LDL* low density lipoprotein, *MI* myocardial infarction, *PCI* percutaneous coronary intervention.

^a^*p* < 0.05 vs. Normal weight.

^b^*p* < 0.05 vs. Overweight.

^c^*p* < 0.05 vs. Obesity.

### Conventional 2D and speckle-tracking echocardiography-derived parameters according to BMI groups

Conventional 2D echocardiographic parameters of patients in the three weight groups are shown in Table 4. Individuals in the group with obesity exhibited higher values of LV end-diastolic dimensions, and LVMi, whereas patients with overweight had higher LV EDVi. Interestingly, LVEF was significantly lower in the normal group compared to the groups with overweight and obesity. Regarding diastolic function, E/A ratio was significantly lower along the increasing weight groups, whereas E/e’ average ratio showed higher values with each weight groups. Regarding STE-derived indices, LVGLS showed a progressive decline across the three weight groups, with normal-weight individuals having the highest absolute LVGLS values and patients with obesity having the lowest. Concerning MW indices, GWI and GCW values were significantly lower in the group with obesity, while there was no difference between the group with normal-weight and overweight. Conversely, GWW and GWE values were comparable between the weight groups. (Table 4) Furthermore, we have compared patients with and without outcomes within the three weight groups (Supplementary Table 2). LVEF was similar between alive and deceased patients in all weight groups. Concerning STE-derived metrics, LVGLS was lower in patients who experienced adverse outcomes in both groups with normal weight and overweight, however, this was not present in individuals with obesity. Conversely, GWI values were significantly lower in patients with adverse outcomes but only among the group with obesity (Supplementary Table 2).

**Table 4:** Echocardiographic parameters of study population according to different BMI groups.

	Overall (*n* = 1330)	Normal weight (*n* = 405)	Overweight (*n* = 526)	Obesity (*n* = 399)	*p* value
2D conventional echocardiographic data					
LVIDd, mm	48.29 ± 4.79	46.02 ± 4.07^bc^	48.68 ± 4.50^ac^	50.17 ± 4.93^ab^	**<0.001**
IVSd, mm	9.94 ± 1.75	9.10 ± 1.50^bc^	10.09 ± 1.68^ac^	10.62 ± 1.74^ab^	**<0.001**
PWd, mm	9.37 ± 1.51	8.57 ± 1.36^bc^	9.58 ± 1.43^ac^	9.92 ± 1.42^ab^	**<0.001**
RWT, %	0.44 ± 0.13	0.41 ± 0.12^bc^	0.46 ± 0.14^a^	0.45 ± 0.14^a^	**<0.001**
LVMi, g/m^2^	87.50 ± 21.52	79.20 ± 18.43^bc^	89.79 ± 21.74^a^	93.14 ± 21.71^a^	**<0.001**
LV ESVi, ml/m^2^	20.63 ± 4.98	20.30 ± 5.11	21.06 ± 5.09	20.41 ± 4.68	**0.041**
LV EDVi, ml/m^2^	59.15 ± 11.09	57.00 ± 10.65^bc^	60.62 ± 11.39^a^	59.40 ± 10.81^a^	**<0.001**
LVEF, %	65.11 ± 5.21	64.44 ± 5.14^bc^	65.29 ± 5.08^a^	65.54 ± 5.41^a^	**0.007**
E, cm/s	76.40 ± 17.69	80.80 ± 17.32^bc^	73.55 ± 16.22^a^	75.76 ± 19.02^a^	**<0.001**
A, cm/s	68.88 ± 21.00	61.00 ± 19.52^bc^	67.88 ± 19.87^ac^	77.93 ± 20.45^ab^	**<0.001**
E/A	1.22 ± 0.51	1.47 ± 0.60^bc^	1.18 ± 0.47^ac^	1.03 ± 0.36^ab^	**<0.001**
DT (ms)	201.89 ± 58.58	188.52 ± 56.87^bc^	205.01 ± 57.32^a^	210.93 ± 59.68^a^	**<0.001**
Mitral lateral s’, cm/s	9.88 ± 2.69	10.60 ± 2.73^bc^	9.66 ± 2.62^a^	9.45 ± 2.60^a^	**<0.001**
Mitral lateral e’, cm/s	12.31 ± 4.17	14.48 ± 4.34^bc^	11.93 ± 4.07^ac^	10.63 ± 3.06^ab^	**<0.001**
Mitral lateral a’, cm/s	10.39 ± 3.18	9.36 ± 2.99^bc^	10.58 ± 3.08^ac^	11.17 ± 3.21^ab^	**<0.001**
Mitral medial s’, cm/s	8.58 ± 1.79	8.80 ± 1.84^c^	8.61 ± 1.72^c^	8.31 ± 1.79^ab^	**<0.001**
Mitral medial e’, cm/s	9.80 ± 3.38	11.65 ± 3.62^bc^	9.46 ± 3.11^ac^	8.36 ± 2.53^ab^	**<0.001**
Mitral medial a’, cm/s	10.46 ± 2.42	9.75 ± 2.66^bc^	10.88 ± 2.27^a^	10.64 ± 2.17^a^	**<0.001**
E/e’ average	7.47 ± 2.59	6.64 ± 2.44^bc^	7.39 ± 2.39^ac^	8.38 ± 2.70^ab^	**<0.001**
LAVi, ml/m^2^	28.94 ± 9.88	26.67 ± 9.21^bc^	29.35 ± 9.58^a^	30.70 ± 10.49^a^	**<0.001**
RVd, mm	34.62 ± 5.41	33.20 ± 4.61^bc^	35.06 ± 5.72^a^	35.50 ± 5.47^a^	**<0.001**
RAVi, ml/m^2^	23.13 ± 7.88	23.15 ± 7.94	23.65 ± 7.99	22.40 ± 7.62	0.069
TAPSE, mm	23.85 ± 4.04	24.00 ± 4.07	23.99 ± 3.89	23.51 ± 4.17	0.149
Speckle tracking echocardiography data					
LVGLS, %	−19.94 ± 3.49	−20.86 ± 3.55^bc^	−19.95 ± 3.33^ac^	−18.99 ± 3.39^ab^	**<0.001**
Myocardial work data					
GWI, mmHg%	2040.65 ± 483.64	2062.04 ± 489.65^c^	2076.51 ± 468.3^c^	1971.66 ± 491.4^ab^	**0.003**
GCW, mmHg%	2194.21 ± 472.40	2227.67 ± 466.36^c^	2236.33 ± 463.3^c^	2104.74 ± 479.3^ab^	**<0.001**
GWW, mmHg%	144.97 ± 104.14	146.78 ± 106.50	147.37 ± 104.63	139.97 ± 101.11	0.517
GWE, %	93.72 ± 4.27	93.75 ± 4.29	93.73 ± 4.22	93.66 ± 4.31	0.952

Continuous variables are presented as means ± SD. Bold: significant *p* values.

*A* atrial contraction, *a’* peak late (atrial) diastolic annular velocity, *DT* deceleration time, *E* early diastolic filling, *e’* early diastolic annular velocity, *EDVi* end diastolic volume index, *EF* ejection fraction, *ESVi* end-systolic volume index, *GCW* global constructive work, *GLS* global longitudinal strain, *GWE* global work efficiency, *GWI* global work index, *GWW* global wasted work, *IVSd* inter-ventricular septal diameter, *LAVi* left atrial volume index, *LV* left ventricle, *LVIDd* left ventricular internal diameter at end-diastole, *LVMi* left ventricular mass index, *PWd* posterior wall diameter, *RVd* right ventricle diameter, *RWT* relative wall thickness, *s’* systolic annular velocity, *TAPSE* tricuspid annular plane systolic excursion.

^a^*p* < 0.05 vs. Normal weight.

^b^*p* < 0.05 vs. Overweight.

^c^*p* < 0.05 vs. Obesity.

### Long-term prognostic value of LV systolic function in different weight groups

We have performed univariable Cox regression analysis in the total cohort and within the three different weight groups (Table 5, Supplementary Tables 3–6). Focusing on the prognostic value of LV systolic function; in the total cohort, LVEF was not associated with the adverse outcome, whereas LVGLS, GWI, GWE, and GWW were significant predictors of all-cause mortality (Table 5, Supplementary Table 3). Conversely, when assessing the normal weight group only LVGLS was a predictor of the adverse outcome, whereas LVEF or MW metrics were not (Table 5, Supplementary Table 4). In the group with overweight, LVGLS, along with GWE and GWW, were significant predictors, whereas LVEF, GWI, and GCW were not (Table 4, Supplementary Table 5). Finally, in subjects with obesity, only GWI emerged as a significant predictor of all-cause mortality (Table 5, Supplementary Table 6). To adjust for potential clinical cofounders, multivariable Cox regression models were built based on clinical differences observed in Table 3. When adjusting for female sex, BMI, and systolic blood pressure, GWI still remained an independent significant predictor of all-cause mortality in the total cohort, and in patients with overweight and obesity, but interestingly not in patients with normal weight (Table 5).

**Table 5:** Association of echocardiography-derived LV systolic function metrics with all-cause mortality in different weight groups.

Univariable Cox regressions in different subgroups								
	Total Cohort (*n* = 1330)		Normal weight (*n* = 405)		Overweight (*n* = 526)		Obesity (*n* = 399)	
	HR [95% CI]	*p* value	HR [95% CI]	*p* value	HR [95% CI]	*p* value	HR [95% CI]	*p* value
LVEF	0.971 [0.941–1.002]	0.066	0.966 [0.892–1.045]	0.385	0.961 [0.918–1.007]	0.093	0.972 [0.924–1.021]	0.259
LVGLS	1.100 [1.049–1.154]	**<0.001**	1.156 [1.031–1.295]	**0.013**	1.089 [1.014–1.170]	**0.020**	1.051 [0.967–1.141]	0.239
GWE	0.947 [0.914–0.981]	**0.002**	1.012 [0.915–1.119]	0.823	0.917 [0.874–0.963]	**<0.001**	0.964 [0.906–1.025]	0.238
GWI	0.958 [0.924–0.992]	**0.017**	1.001 [0.919–1.090]	0.984	0.976 [0.917–1.019]	0.212	0.929 [0.875–0.986]	**0.015**
GCW	0.979 [0.944–1.015]	0.243	1.002 [0.916–1.096]	0.970	1.003 [0.952–1.058]	0.902	0.943 [0.887–1.003]	0.064
GWW	1.194 [1.041–1.369]	**0.011**	0.938 [0.618–1.424]	0.764	1.341 [1.121–1.604]	**0.001**	1.109 [0.836–1.425]	0.419
Multivariable Cox regressions in different subgroups								
Female sex	0.517 [0.367–0.728]	**<0.001**	0.224 [0.091–0.553]	**0.001**	0.679 [0.411–1.120]	0.129	0.600 [0.335–1.075]	0.086
SBP	1.029 [1.019–1.038]	**<0.001**	1.049 [1.030–1.067]	**<0.001**	1.028 [1.014–1.041]	**<0.001**	1.012 [0.993–1.032]	0.209
BMI	1.016 [0.981–1.053]	0.365	0.832 [0.678–1.022]	0.080	0.938 [0.783–1.122]	0.482	1.027 [0.946–1.116]	0.526
GWI	**0.923 [0.889–0.959]**	**<0.001**	0.934 [0.856–1.019]	0.122	**0.922 [0.872–0.975]**	**0.005**	**0.920 [0.863–0.981]**	**0.011**

Bold: significant *p* values.

*BMI* body mass index, *EF* ejection fraction, *GCW* global constructive work, *GWE* global work efficiency, *GWI* global work index, *GWW* global wasted work, *LV* left ventricle, *LVGLS* left ventricular global longitudinal strain, *SBP* systolic blood pressure.

**Fig. 1:**
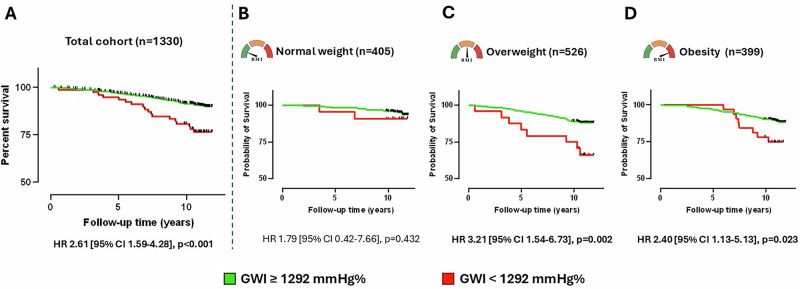
Kaplan–Meier survival curves using the previously published cut-off value of 1292 mmHg% in the total cohort and in the different BMI groups. Total cohort (GWI ≥ 1292 Hgmm—*n* = 1252, GWI < 1292 Hgmm%—*n* = 78) (**A**), groups with normal weight (GWI ≥ 1292 Hgmm—*n* = 383, GWI < 1292 Hgmm%—*n* = 22) (**B**), overweight (GWI ≥ 1292 Hgmm—*n* = 502, GWI < 1292 Hgmm%—*n* = 24) (**C**) and obesity (GWI ≥ 1292 Hgmm—*n* = 367, GWI < 1292 Hgmm%—*n* = 32) (**D**).

Furthermore, participants were dichotomized based on a previously established GWI cut-off value of 1292 mmHg% [27]. Applying this threshold, GWI effectively differentiated between high-risk and low-risk groups in terms of all-cause mortality in the total cohort, and in the subgroups with overweight and obesity, but not in the groups with normal weight (Fig. 1). As the Kaplan–Meier survival curves indicate, those subjects with overweight and GWI values below 1292 mmHg% experienced more than 3-fold higher risk of all-cause mortality (Fig. 1C). Similarly, in the subgroup with obesity (Fig. 1D) and in the total cohort (Fig. 1A), participants with GWI values below the cut-off had more than 2-times higher risk for adverse events. Furthermore, multivariable Cox proportional hazard models were also built adjusting for the previously used confounders (female sex SBP, BMI). Even after adjusting for relevant clinical variables, the guideline-based GWI cutoff was independently associated with the outcome, as patients with GWI values below 1292 mmHg% experienced a higher risk of all-cause mortality in the total cohort and in all weight groups, respectively (Supplementary Table 7).

## Discussion

To the best of our knowledge, this is the first study that investigated the long-term prognostic role of MW indices in individuals with overweight and obesity, in a low-risk community-based cohort. We demonstrated that GWI was an independent significant predictor of all-cause mortality in individuals with obesity, whereas GWW and GWE were associated with adverse outcomes in individuals with overweight. Interestingly, LVEF did not have prognostic significance in these cohorts. Furthermore, by utilizing the previously established cut-off GWI value of 1292 mmHg% [27], we were able to effectively differentiate low and high risk groups even after adjusting to clinically relevant confounders, as the risk of all-cause mortality was more than four-times higher in patients with overweight and more than two times higher in the participant with obesity.

Amid the growing prevalence of obesity and its cardiovascular implications, clinical and research initiatives have increasingly focused on risk stratification of individuals with obesity. Echocardiography is a cost-effective, non-invasive, and reliable diagnostic tool, offering added value in routine cardiovascular risk assessment for populations with overweight and obesity. Assessment of cardiac function has great significance, as obesity is strongly linked to structural, functional, and hemodynamic changes that play a pivotal role in the development of cardiovascular diseases [28–31]. It is also well recognized that in individuals with overweight or obesity increased blood volume, cardiac output, and systemic vascular resistance, combined with the activation of the renin-angiotensin-aldosterone system and heightened sympathetic activity, lead to adverse cardiac remodeling and deteriorating cardiac function [31–33].

Various studies have shown that obesity leads to LV dilation, increased LV volumes, mass index, and relative wall thickness [14, 34] similarly, to our findings. Chinali et al. also reported that obesity, in addition to LV geometric abnormalities, was associated with reduced LVEF [14]. Although conventional echocardiographic parameters, such as LVEF, remain standard for assessing systolic function in individuals with obesity [16, 17, 35, 36], more advanced techniques such as LVGLS have garnered significant attention for detecting subclinical systolic dysfunction, particularly when LVEF remained within normal ranges [16, 17, 37, 38]. In a multicenter, cross-sectional study, Snelder et al. identified a high prevalence of subclinical cardiac dysfunction among individuals with obesity without known cardiovascular disease [37]. This dysfunction was most reliably detected by LVGLS and was linked to autonomic dysregulation rather than to traditional cardiovascular risk factors [37]. Our study verifies these findings in a large cohort of low-risk community-based cohort, as LVEF remained similarly in normal ranges throughout all weight subgroups, whereas LVGLS showed a gradual decline with increasing BMI. However, despite the gradual decline in LVGLS, its prognostic value was limited in individuals with obesity. The prognostic performance of GWI in individuals with obesity—particularly when conventional systolic parameters such as LVEF and LVGLS remain relatively unchanged—suggests that MW analysis may serve as an additional tool in clinical risk assessment. Rather than replacing standard measures, it could complement them by unmasking early or subtle dysfunction in patients with underlying cardiometabolic deterioration who otherwise appear low-risk.

In our study, we observed that individuals with overweight exhibited similar GWI and GCW values as normal-weight counterparts, while LVGLS showed a notable decline in the group with overweight. This discrepancy likely reflects the hemodynamic and neurohormonal alterations associated with elevated BMI, including augmented preload and afterload and sympathetic activation, all of which promote increased LV contractility and work, as well as elevated blood pressure. In our findings, individuals with overweight had significantly higher systolic and diastolic blood pressure values compared to patients in the normal-weight group, yet MW indices were similar between the two groups, likely explained by that MW analysis takes afterload into account. This; however, may not necessarily be taken as a marker of healthy cardiac function. Conversely, individuals with obesity demonstrated significantly lower GWI and GCW compared to both groups with overweight and normal weight, likely attributable to adverse LV remodeling and myocardial fibrosis associated with the obesity stage. Our findings align with previous studies that demonstrated similar patterns of MW changes, characterized by reductions in GCW and GWI, independent of systolic blood pressure [35, 39]. Initially, elevated preload, afterload, and sympathetic activation result in maintained or even increased LV work and contractility, but over time, these adaptive mechanisms fail, leading to maladaptive changes such as fibrosis, inflammation, and metabolic dysfunction, adverse LV remodeling, and ultimately resulting in a marked reduction in GWI.

In the context of risk assessment in a low-risk population, studies have shown, that STE-derived parameters such as LVGLS and peak atrial longitudinal strain have shown significant predictive value [11–13, 36]. However, evidence is scarce regarding the long-term predictive value of these STE-derived systolic function metrics in individuals with obesity and overweight. In our study, LVGLS was a significant predictor of the primary endpoint in individuals with normal-weight and overweight, but not among participants with obesity. In the group with overweight, both GWW and GWE were significant predictors of the endpoint. This may reflect early metabolic derangements, where enhanced oxidative stress, and inflammation in the myocardial tissue result in ineffective contraction, thus increasing wasted work, and leading to impaired myocardial efficiency. Additionally, elevated LVMi may contribute to the increased GWW observed in participants with overweight, a finding consistent with prior studies [40, 41]. Interestingly, in the group with obesity, GWI was the only parameter of systolic function that predicted all-cause mortality. This may be partially explained by hemodynamic changes observed in obesity, potentially hindering the interpretation of conventional systolic markers such as GLS. Specifically, obesity is markedly associated with an expanded volumetric state and increased venous return, resulting in elevated preload (which increases myocardial deformation), while concomitant arterial stiffening and increased systemic vascular resistance may contribute to chronically increased afterload (which decreases myocardial deformation) [42]. Due to this altered hemodynamics with opposing forces and the load-dependency of deformation metrics, GLS may be unable to detect subclinical changes, which could attenuate its prognostic utility in patients with obesity. Conversely, GWI integrates information from two distinct domains—myocardial deformation and systemic load—which could capture the cumulative burden associated with myocardial impairment (e.g., interstitial fibrosis, altered LV geometry, and contractility) and hemodynamic burden (e.g., stiffening, altered ventriculo-arterial coupling) arising from obesity. Thus MW metrics may better portray the interplay between altered myocardial mechanics and systemic load and serve as an integrative marker showing increased cumulative risk associated with obesity.

While recent studies, such as Olsen et al.'s, have explored the prognostic role of MW indices in the general population, none have specifically addressed their role in different BMI groups within a low-risk cohort [40]. Olsen’s study showed that GWI, GCW, and GWE were associated with adverse outcomes in hypertensive individuals, but did not assess the impact of obesity or overweight [40]. Our results provide an added dimension of clinical relevance by demonstrating the importance of MW assessment in populations with overweight and obesity.

Importantly, although our findings contribute to the growing evidence supporting the prognostic relevance of MW, still the clinical application and integration of MW analysis into routine echocardiographic evaluation warrants further consideration. Focusing on everyday clinical practical perspectives, GWI could be derived from two domains that are routinely acquired in standard echocardiographic protocols—STE-derived GLS quantification and noninvasive brachial blood pressure measurement. Furthermore, MW analysis can be performed in real-time, directly on most modern echocardiographic platforms using integrated, semi-automated software, without requiring additional imaging, time burden, or extensive post-processing (although a fully automated pipeline is currently only commercially available with one vendor). Still, rather than serving as a standalone screening tool, MW analysis may complement more conventional metrics such as LVEF and LVGLS by unmasking subclinical deterioration and may reveal the cumulative burden of hemodynamic inefficiency or reduced contractile reserve in individuals with elevated cardiometabolic risk. Interpreting alongside demographics, clinical risk factors, and routine 2D echocardiographic metrics, MW may support individualized risk stratification and guide closer follow-up, preventive strategies, or early interventions, especially in patients with overweight and obesity.

## Strengths and limitations

One of the major strengths of our study is the fairly large sample derived from a community-based screening program involving subjects from a low-risk cohort. Of note, we were able to conduct MW analysis in a large cohort of individuals with varying stages of obesity. It is also important to highlight that we had fairly large and evenly distributed sample sizes in all three BMI subgroups. Lastly, an important strength is the long follow up time enabling to assess long-term prognosis. Common limitations are also have to be stated. This is a retrospective, single-center study, which may affect the generalizability and interpretation of the findings. Although only apical-four chamber views were utilized for the quantification of LV systolic function, this approach has been approved and validated in previous studies [43, 44]. While this study is the first to demonstrate the prognostic power of MW metrics in individuals with overweight and obesity in the general population, further prospective and multi-center studies are required to validate and strengthen these results.

## Conclusion

MW analysis-derived metrics were found to be robust, independent predictors of all-cause mortality in low-risk individuals with different stages of obesity. These findings underscore the limitations of conventional echocardiographic measures, which may underestimate cardiovascular risk in populations with overweight and obesity, highlighting the potential of MW analysis to refine risk stratification and improve prognostic accuracy in this growing patient cohort.

## Supplementary information

Supplementary material

## Data Availability

The de-identified data underlying this article will be shared for non-commercial purposes, without breaching participant confidentiality on reasonable request to the corresponding author.
