# Supplementary material for: Long-term prognostic value of myocardial work analysis across obesity stages: insights from a community-based study
Source: Int J Obes (Lond). 2025 Aug 6;49(10):2032–41. doi: 10.1038/s41366-025-01863-w (PMC12532703; doi:10.1038/s41366-025-01863-w)
Supplement: Supplementary file 1 — Supplementary material [file 41366_2025_1863_MOESM1_ESM.docx]

**SUPPLEMENTARY TABLES**

| **Supplementary table 1. Demographic and clinical characteristics of the study samples according to different weight groups and primary outcome** | | | | | | | | | |
| --- | --- | --- | --- | --- | --- | --- | --- | --- | --- |
|  | **Normal weight (n=405)** | | | **Overweight (n=526)** | | | **Obesity (n=399)** | | |
|  | **Alive** | **Deceased** | **p** | **Alive** | **Deceased** | **p** | **Alive** | **Deceased** | **p** |
| **Baseline demographic characteristics** | | | | | | | | | |
| **Age (years)** | 45.85±13.88 | 61.15±24.45 | **<0.001** | 52.92±13.66 | 66.94±14.63 | **<0.001** | 57.18±11.45 | 68.10±10.09 | **<0.001** |
| **Female, n (%)** | 280 (73.11) | 10 (45.45) | **0.005** | 256 (48.95) | 42 (37.31) | **0.144** | 132 (62.29) | 25 (48.98) | **0.074** |
| **Clinical characteristics** | | | | | | | | | |
| **BSA, m^2^** | 1.72±0.17 | 1.68±0.24 | **0.248** | 1.93±0.18 | 1.88±0.13 | **0.015** | 2.07±0.19 | 2.04±0.22 | **0.491** |
| **BMI, kg/m^2^** | 22.37±1.89 | 22.28±2.17 | **0.834** | 27.41±1.38 | 27.37±1.45 | **0.821** | 33.76±3.40 | 34.20±2.98 | **0.394** |
| **Systolic blood pressure, mmHg** | 125.25±18.29 | 143.66±27.84 | **<0.001** | 133.34±16.99 | 140.00±21.58 | **0.004** | 137.63±15.91 | 138.57±16.27 | **0.700** |
| **Diastolic blood pressure, mmHg** | 77.05±9.92 | 78.57±12.47 | **0.492** | 79.43±8.92 | 77.91±9.98 | **0.200** | 82.68±9.13 | 76.51±9.38 | **<0.001** |
| **Heart rate, bpm** | 66.97±9.76 | 63.95±9.80 | **0.169** | 67.37±9.94 | 68.89±9.02 | **0.241** | 69.76±9.74 | 71.10±9.36 | **0.369** |
| **Risk factors and medical history** | | | | | | | | | |
| **Smoking history, n (%)** | 154 (40.21) | 7 (31.82) | **0.434** | 182 (39.65) | 31 (46.27) | **0.303** | 158 (45.14) | 25 (51.02) | **0.439** |
| **Hypertension, n (%)** | 167 (43.60) | 10 (45.45) | **0.865** | 221 (48.15) | 35 (52.24) | **0.531** | 172 (49.14) | 20 (40.82) | **0.275** |
| **Diabetes, n (%)** | 44 (11.49) | 3 (13.64) | **0.760** | 62 (13.51) | 4 (5.97) | **0.082** | 52 (14.86) | 4 (8.16) | **0.206** |
| **Arrhythmia, n (%)** | 44 (11.49) | 1 (4.55) | **0.314** | 43 (9.37) | 7 (10.45) | **0.778** | 35 (10.00) | 8 (16.33) | **0.181** |
| **Previous MI, n (%)** | 10 (2.61) | 0 (0.00) | **0.443** | 9 (1.96) | 0 (0.00) | **0.248** | 19 (5.43) | 1 (2.04) | **0.309** |
| **Previous PCI, n (%)** | 6 (1.57) | 0 (0.00) | **0.554** | 11 (2.40) | 1 (1.49) | **0.643** | 6 (1.71) | 0 (0.00) | **0.356** |
| **Previous CABG, n (%)** | 6 (1.57) | 0 (0.00) | **0.554** | 3 (0.65) | 1 (1.49) | **0.460** | 2 (0.57) | 0 (0.00) | **0.596** |
| **Chronic heart failure, n (%)** | 29 (7.57) | 0 (0.00) | **0.180** | 31 (6.75) | 2 (2.99) | **0.235** | 21 (6.00) | 0 (0.00) | **0.078** |
| **Previous stroke, n (%)** | 15 (3.92) | 0 (0.00) | **0.344** | 29 (6.32) | 3 (4.48) | **0.556** | 14 (4.00) | 1 (2.04) | **0.499** |
| **Pulmonary disease, n (%)** | 22 (5.74) | 1 (4.55) | **0.814** | 34 (7.41) | 7 (10.45) | **0.386** | 28 (8.00) | 5 (10.20) | **0.600** |
| **Laboratory work** | | | | | | | | | |
| **Total cholesterol,mmol/L** | 5.57±1.15 | 5.52±0.77 | **0.455** | 5.45±1.15 | 5.72±1.16 | **0.070** | 5.42±1.11 | 5.37±0.98 | **0.777** |
| **HDL cholesterol, mmol/L** | 1.45±0.43 | 1.51±0.38 | **0.773** | 1.49±0.46 | 1.51±0.48 | **0.656** | 1.44±0.41 | 1.56±0.39 | **0.049** |
| **LDL cholesterol, mmol/L** | 3.46±0.97 | 3.37±.72 | **0.666** | 3.33±0.99 | 3.60±0.94 | **0.052** | 3.32±0.97 | 3.18±0.88 | **0.359** |
| **Triglycerides, mmol/L** | 2.32±1.63 | 2.10±1.31 | **0.202** | 2.24±1.74 | 2.28±1.52 | **0.870** | 2.29±1.45 | 2.04±1.64 | **0.279** |
| **Glucose, mmol/L** | 6.05±1.69 | 5.82±0.98 | **0.495** | 5.93±1.36 | 5.85±1.23 | **0.642** | 6.05±1.64 | 5.65±0.94 | **0.097** |
| **ProBNP, pmol/L** | 135.33±449.12 | 141.96±246.05 | **0.559** | 122.31±217.51 | 92.11±108.30 | **0.269** | 135.45±318.86 | 92.39±97.74 | **0.354** |
| **Serum Creatinine, µmol/L** | 77.74±16.85 | 77.83±16.44 | **0.910** | 77.30±20.88 | 75.85±19.75 | **0.595** | 77.17±17.59 | 76.39±16.06 | **0.767** |
| **HBa1c, %** | 5.76±0.75 | 5.75±0.58 | **0.463** | 5.77±0.75 | 5.62±0.45 | **0.124** | 5.74±0.74 | 5.52±0.47 | **0.048** |
| *Continuous variables are presented as means ± SD, categorical variables are reported as frequencies (%); BMI, body mass index; BSA, body surface area; CABG, coronary artery bypass grafting; HDL, high density lipoprotein; LDL, low density lipoprotein; MI, myocardial infarction; PCI, percutaneous coronary intervention* | | | | | | | | | |

| **Supplementary table 2. Echocardiographic parameters of study population according to different weight groups and primary outcome** | | | | | | | | | |
| --- | --- | --- | --- | --- | --- | --- | --- | --- | --- |
|  | **Normal weight (n=405)** | | | **Overweight (n=526)** | | | **Obesity (n=399)** | | |
|  | **Alive** | **Deceased** | **p** | **Alive** | **Deceased** | **p** | **Alive** | **Deceased** | **p** |
| **2D conventional echocardiographic data** | | | | | | | | | |
| **LVIDd, mm** | 45.93±4.02 | 47.41±4.60 | **0.097** | 48.59±4.45 | 49.36±4.86 | **0.222** | 50.01±4.83 | 51.50±5.54 | **0.084** |
| **IVSd, mm** | 9.05±1.42 | 10.00±2.27 | **0.004** | 10.02±1.62 | 10.67±1.99 | **0.005** | 10.49±1.65 | 11.62±2.05 | **<0.001** |
| **PWd, mm** | 8.57±1.36 | 8.68±1.32 | **0.701** | 9.52±1.37 | 10.02±1.79 | **0.014** | 9.85±1.35 | 10.40±1.80 | **0.018** |
| **RWT, %** | 0.41±0.12 | 0.37±0.06 | **0.135** | 0.45±0.14 | 0.49±0.16 | **0.042** | 0.44±0.13 | 0.47±0.16 | **0.186** |
| **LVMi, g/m^2^** | 78.31±17.16 | 94.32±30.18 | **<0.001** | 88.21±20.21 | 101.60±28.44 | **<0.001** | 91.22±20.37 | 107.60±25.95 | **<0.001** |
| **LV ESVi, ml/m^2^** | 20.18±4.98 | 22.54±6.78 | **0.039** | 20.89±5.05 | 22.20±5.20 | **0.049** | 20.19±4.52 | 22.03±5.46 | **0.009** |
| **LV EDVi, ml/m^2^** | 56.73±10.39 | 62.03±14.06 | **0.026** | 60.39±11.64 | 62.15±9.38 | **0.239** | 58.96±10.72 | 62.50±11.09 | **0.032** |
| **LVEF, %** | 64.49±5.07 | 63.53±6.29 | **0.394** | 65.43±4.96 | 64.30±5.76 | **0.088** | 65.66±5.32 | 64.74±6.04 | **0.267** |
| **E, cm/s** | 80.92±16.85 | 78.42±25.25 | **0.540** | 74.36±15.88 | 67.84±17.57 | **0.003** | 75.36±17.39 | 78.59±28.05 | **0.265** |
| **A, cm/s** | 60.72±19.08 | 66.53±26.75 | **0.206** | 66.20±18.97 | 79.56±22.15 | **<0.001** | 76.70±20.13 | 86.55±20.74 | **0.002** |
| **E/A** | 1.47±0.57 | 1.50±1.039 | **0.801** | 1.22±0.46 | 0.92±0.39 | **<0.001** | 1.04±0.35 | 0.96±0.45 | **0.149** |
| **DT (ms)** | 186.29±53.63 | 231.79±92.98 | **<0.001** | 201.14±53.04 | 232.08±76.38 | **<0.001** | 208.10±59.49 | 230.92±57.77 | **0.012** |
| **Mitral lateral s', cm/s** | 10.63±2.71 | 10.09±3.13 | **0.369** | 9.76±2.59 | 8.99±2.80 | **0.024** | 9.48±2.57 | 9.27±2.83 | **0.594** |
| **Mitral lateral e', cm/s** | 14.67±4.18 | 11.41±5.73 | **<0.001** | 12.24±3.99 | 9.78±3.97 | **<0.001** | 10.76±3.05 | 9.67±2.97 | **0.019** |
| **Mitral lateral a', cm/s** | 9.34±2.96 | 9.64±3.65 | **0.653** | 10.41±2.99 | 11.75±3.45 | **<0.001** | 11.13±3.12 | 11.43±3.84 | **0.540** |
| **Mitral medial s', cm/s** | 8.81±1.79 | 8.55±2.58 | **0.508** | 8.69±1.66 | 8.05±2.00 | **0.004** | 8.37±1.79 | 7.90±1.72 | **0.086** |
| **Mitral medial e', cm/s** | 11.79±3.54 | 9.36±4.23 | **0.002** | 9.71±3.03 | 7.74±3.08 | **<0.001** | 8.49±2.55 | 7.41±2.16 | **0.005** |
| **Mitral medial a', cm/s** | 9.77±2.59 | 9.41±3.69 | **0.535** | 10.85±2.21 | 11.75±3.45 | **0.518** | 10.68±2.14 | 10.35±2.37 | **0.309** |
| **E/e' average** | 6.48±1.98 | 9.82±6.01 | **<0.001** | 7.23±2.17 | 8.51±3.37 | **<0.001** | 8.21±2.50 | 9.63±3.63 | **<0.001** |
| **LAVi, ml/m^2^** | 26.26±8.62 | 33.97±15.26 | **<0.001** | 28.83±9.01 | 32.96±12.31 | **0.001** | 30.17±10.34 | 34.43±10.95 | **0.008** |
| **RVd, mm** | 33.18±4.58 | 33.45±5.32 | **0.788** | 34.96±5.68 | 35.71±5.94 | **0.319** | 35.45±5.46 | 35.88±5.60 | **0.616** |
| **RAVi, ml/m^2^** | 23.00±7.97 | 26.00±6.99 | **0.099** | 23.34±5.68 | 25.84±9.21 | **0.021** | 22.25±7.62 | 23.59±7.56 | **0.271** |
| **TAPSE, mm** | 24.11±4.08 | 22.05±3.59 | **0.027** | 23.86±3.81 | 24.88±4.40 | **0.057** | 23.44±4.13 | 23.98±4.51 | **0.420** |
| **Speckle tracking echocardiography data** | | | | | | | | | |
| **LVGLS, %** | -20.96±3.44 | -19.10±4.90 | **0.017** | -20.08±3.24 | -19.05±3.80 | **0.017** | -19.06±3.41 | -18.46±4.11 | **0.242** |
| **Myocardial work data** | | | | | | | | | |
| **GWI, mmHg%** | 2061.73±486.49 | 2067.42±554.20 | **0.958** | 2086.42±449.05 | 2008.64±582.94 | **0.204** | 1992.89±486.29 | 1820.07±505.12 | **0.021** |
| **GCW, mmHg%** | 2227.35±484.08 | 2233.22±503.49 | **0.954** | 2235.67±447.82 | 2240.82±562.05 | **0.932** | 2119.50±472.87 | 1990.30±515.89 | **0.100** |
| **GWW, mmHg%** | 147.17±106.67 | 139.94±105.64 | **0.757** | 141.73±100.65 | 186.06±122.67 | **0.001** | 138.33±100.31 | 151.71±107.03 | **0.386** |
| **GWE, %** | 93.74±4.31 | 93.96±4.02 | **0.814** | 93.97±4.01 | 92.04±5.20 | **<0.001** | 93.76±4.25 | 92.98±4.70 | **0.239** |
| *Continuous variables are presented as means ± SD; A, atrial contraction; a’, peak late (atrial) diastolic annular velocity; DT, deceleration time; E, early diastolic filling; e’, early diastolic annular velocity; EDVi, end diastolic volume index; EF, ejection fraction; ESVi, end-systolic volume index; GCW, global constructive work; GLS, global longitudinal strain; GWE, global work efficiency; GWI, global work index; GWW, global wasted work; IVSd, inter-ventricular septal diameter; LAVi, left atrial volume index; LV, left ventricle; LVIDd, left ventricular internal diameter at end-diastole; LVMi, left ventricular mass index; PWd, posterior wall diameter; RVd, right ventricle diameter; RWT, relative wall thickness; s’, systolic annular velocity; TAPSE, tricuspid annular plane systolic excursion* | | | | | | | | | |

**Supplementary Table 3. Factors associated with all-cause mortality using univariable Cox regression in the total cohort**

| **Univariable Cox regression** | | |
| --- | --- | --- |
|  | **HR [95% CI]** | **p-value** |
| Age | 1.089 [1.073 - 1.107] | <0.001 |
| Female sex | 0.516 [0.369–0.724] | <0.001 |
| Systolic blood pressure | 1.020 [1.012–1.027] | <0.001 |
| BMI | 1.048 [1.016 - 1.081] | 0.003 |
| LVIDd | 1.066 [1.028 - 1.104] | <0.001 |
| IVSd | 1.298 [1.200 - 1.403] | <0.001 |
| PWd | 1.254 [1.131 - 1.390] | <0.001 |
| RWT | 3.408 [1.046 – 11.110] | 0.042 |
| LV Mi | 1.027 [1.021 - 1.033] | <0.001 |
| LV ESVi | 1.062 [1.030 - 1.094] | <0.001 |
| LV EDVi | 1.024 [1.011 - 1.038] | <0.001 |
| LVEF | 0.971 [0.941 - 1.002] | 0.066 |
| E | 0.989 [0.979 – 0.999] | 0.031 |
| A | 1.023 [1.016 - 1.029] | <0.001 |
| E/A | 0.307 [0.190 - 0.496] | <0.001 |
| DT | 1.008 [1.005 - 1.010] | <0.001 |
| Mitral lateral s*'* | 0.905 [0.846 – 0.969] | 0.004 |
| Mitral lateral e*'* | 0.842 [0.801 - 0.884] | <0.001 |
| Mitral lateral a*'* | 1.095 [1.043 - 1.150] | <0.001 |
| Mitral medial s*'* | 0.824 [0.742 – 0.914] | <0.001 |
| Mitral medial e' | 0.792 [0.742 - 0.845] | <0.001 |
| Mitral medial a*'* | 1.013 [0.945 - 1.087] | 0.711 |
| E/e*'* average | 1.195 [1.144 - 1.248] | <0.001 |
| LAVi | 1.045 [1.030 - 1.060] | <0.001 |
| RVd | 1.028 [0.997 - 1.060] | 0.079 |
| TAPSE | 1.017 [0.974 - 1.062] | 0.452 |
| RAVi | 1.030 [1.010 - 1.050] | 0.003 |
| LVGLS | 1.100 [1.049 - 1.154] | <0.001 |
| GWE | 0.947 [0.914 – 0.981] | 0.002 |
| GWI | 0.958 [0.924 – 0.992] | 0.017 |
| GCW | 0.979 [0.944 – 1.015] | 0.243 |
| GWW | 1.194 [1.041 - 1.369] | 0.011 |

*A, atrial contraction; a’, peak late (atrial) diastolic annular velocity; BMI, body mass index; CI, confidence interval; DT, deceleration time; E, early diastolic filling; e’, early diastolic annular velocity; EDVi, end diastolic volume index; EF, ejection fraction; ESVi, end-systolic volume index; GCW, global constructive work; GWE, global work efficiency; GWI, global work index; GWW, global wasted work; HR, hazard ratio; IMT, intima-media thickness; IVSd, inter-ventricular septal diameter; LAVi, left atrial volume index; LV, left ventricle; LV GLS, left ventricular global longitudinal strain; LVIDd, left ventricular internal diameter at end-diastole; LV Mi, left ventricular mass index; PWd, posterior wall diameter; PACS, peak atrial contraction strain; PALS, peak atrial longitudinal strain; RAVi, right atrial volume index; RVd, right ventricle diameter; RWT, relative wall thickness; s’, systolic annular velocity; TAPSE, tricuspid annular plane systolic excursion*

**Supplementary Table 4. Factors associated with all-cause mortality using univariable Cox regression in the group with normal weight**

| **Univariable Cox regression** | | |
| --- | --- | --- |
|  | **HR [95% CI]** | **p-value** |
| Age | 1.072 [1.040 - 1.105] | <0.001 |
| Female sex | 0.314 [0.136–0.728] | 0.007 |
| Systolic blood pressure | 1.034 [1.019–1.050] | <0.001 |
| BMI | 0.977 [0.782 - 1.221] | 0.840 |
| LVIDd | 1.089 [0.988 - 1.200] | 0.087 |
| IVSd | 1.405 [1.127 - 1.752] | 0.003 |
| PWd | 1.061 [0.792 - 1.423] | 0.690 |
| RWT | 0.022 [0.000 - 3.007] | 0.128 |
| LV Mi | 1.035 [1.017 - 1.052] | <0.001 |
| LV ESVi | 1.076 [1.005 - 1.152] | 0.034 |
| LV EDVi | 1.042 [1.005 - 1.080] | 0.024 |
| LVEF | 0.966 [0.892 - 1.045] | 0.385 |
| E | 0.992 [0.966 - 1.018] | 0.530 |
| A | 1.013 [0.992 - 1.034] | 0.220 |
| E/A | 1.122 [0.530 - 2.375] | 0.764 |
| DT | 1.011 [1.005 - 1.017] | <0.001 |
| Mitral lateral s*'* | 0.928 [0.790 - 1.089] | 0.360 |
| Mitral lateral e*'* | 0.835 [0.753 - 0.927] | 0.001 |
| Mitral lateral a*'* | 1.032 [0.900 - 1.183] | 0.652 |
| Mitral medial s*'* | 0.913 [0.713 - 1.169] | 0.471 |
| Mitral medial e' | 0.814 [0.714 - 0.927] | 0.002 |
| Mitral medial a*'* | 0.940 [0.792 - 1.116] | 0.481 |
| E/e*'* average | 1.269 [1.171 - 1.376] | <0.001 |
| LAVi | 1.074 [1.036 - 1.113] | <0.001 |
| RVd | 1.015 [0.927 - 1.111] | 0.755 |
| TAPSE | 0.871 [0.770 - 0.985] | 0.028 |
| RAVi | 1.036 [0.993 - 1.081] | 0.106 |
| LVGLS | 1.156 [1.031 - 1.295] | 0.013 |
| GWE | 1.012 [0.915 - 1.119] | 0.823 |
| GWI | 1.001 [0.919 - 1.090] | 0.984 |
| GCW | 1.002 [0.916 - 1.096] | 0.970 |
| GWW | 0.938 [0.618 - 1.424] | 0.764 |

*A, atrial contraction; a’, peak late (atrial) diastolic annular velocity; BMI, body mass index; CI, confidence interval; DT, deceleration time; E, early diastolic filling; e’, early diastolic annular velocity; EDVi, end diastolic volume index; EF, ejection fraction; ESVi, end-systolic volume index; GCW, global constructive work; GWE, global work efficiency; GWI, global work index; GWW, global wasted work; HR, hazard ratio; IVSd, inter-ventricular septal diameter; LAVi, left atrial volume index; LV, left ventricle; LV GLS, left ventricular global longitudinal strain; LVIDd, left ventricular internal diameter at end-diastole; LV Mi, left ventricular mass index; PWd, posterior wall diameter; PACS, peak atrial contraction strain; PALS, peak atrial longitudinal strain; RAVi, right atrial volume index; RVd, right ventricle diameter; RWT, relative wall thickness; s’, systolic annular velocity; TAPSE, tricuspid annular plane systolic excursion*

**Supplementary Table 5. Factors associated with all-cause mortality using univariable Cox regression in the group with overweight**

| **Univariable Cox regression** | | |
| --- | --- | --- |
|  | **HR [95% CI]** | **p-value** |
| Age | 1.088 [1.063 – 1.113] | <0.001 |
| BMI | 0.976 [0.820 – 1.162] | 0.785 |
| Female sex | 0.695 [0.423–1.140] | 0.149 |
| Systolic blood pressure | 1.017 [1.005–1.028] | 0.003 |
| LVIDd | 1.039 [0.982 – 1.100] | 0.183 |
| IVSd | 1.197 [1.060 – 1.353] | 0.004 |
| PWd | 1.246 [1.045 – 1.486] | 0.014 |
| RWT | 4.638 [0.940 – 22.884] | 0.060 |
| LV Mi | 1.022 [1.013 – 1.032] | <0.001 |
| LV ESVi | 1.044 [0.999 – 1.090] | 0.054 |
| LV EDVi | 1.011 [0.992 – 1.031] | 0.255 |
| LVEF | 0.961 [0.918 – 1.007] | 0.093 |
| E | 0.975 [0.959 – 0.991] | 0.002 |
| A | 1.030 [1.018 – 1.041] | <0.001 |
| E/A | 0.121 [0.051 – 0.287] | <0.001 |
| DT | 1.008 [1.004 – 1.012] | <0.001 |
| Mitral lateral s*'* | 0.891 [0.806 – 0.984] | 0.023 |
| Mitral lateral e*'* | 0.844 [0.785 – 0.908] | <0.001 |
| Mitral lateral a*'* | 1.130 [1.052 – 1.215] | 0.001 |
| Mitral medial s*'* | 0.791 [0.679 – 0.922] | 0.003 |
| Mitral medial e' | 0.773 [0.697 – 0.857] | <0.001 |
| Mitral medial a*'* | 1.037 [0.933 – 1.154] | 0.499 |
| E/e*'* average | 1.164 [1.082 – 1.252] | <0.001 |
| LAVi | 1.038 [1.016 – 1.060] | 0.001 |
| RVd | 1.021 [0.979 – 1.064] | 0.342 |
| TAPSE | 1.067 [1.000 – 1.139] | 0.050 |
| RAVi | 1.034 [1.005 – 1.063] | 0.020 |
| LVGLS | 1.089 [1.014 – 1.170] | 0.020 |
| GWE | 0.917 [0.874 – 0.963] | <0.001 |
| GWI | 0.976 [0.917 – 1.019] | 0.212 |
| GCW | 1.003 [0.952 – 1.058] | 0.902 |
| GWW | 1.341 [1.121 – 1.604] | 0.001 |

*A, atrial contraction; a’, peak late (atrial) diastolic annular velocity; BMI, body mass index; CI, confidence interval; DT, deceleration time; E, early diastolic filling; e’, early diastolic annular velocity; EDVi, end diastolic volume index; EF, ejection fraction; ESVi, end-systolic volume index; GCW, global constructive work; GWE, global work efficiency; GWI, global work index; GWW, global wasted work; HR, hazard ratio; IVSd, inter-ventricular septal diameter; LAVi, left atrial volume index; LV, left ventricle; LV GLS, left ventricular global longitudinal strain; LVIDd, left ventricular internal diameter at end-diastole; LV Mi, left ventricular mass index; PWd, posterior wall diameter; PACS, peak atrial contraction strain; PALS, peak atrial longitudinal strain; RAVi, right atrial volume index; RVd, right ventricle diameter; RWT, relative wall thickness; s’, systolic annular velocity; TAPSE, tricuspid annular plane systolic excursion*

**Supplementary Table 6. Factors associated with all-cause mortality using univariable Cox regression in the group with obesity**

| **Univariable Cox regression** | | |
| --- | --- | --- |
|  | **HR [95% CI]** | **p-value** |
| Age | 1.104 [1.070 – 1.139] | <0.001 |
| Female sex | 0.591 [0.338–1.036] | 0.066 |
| Systolic blood pressure | 1.003 [0.986–1.021] | 0.731 |
| BMI | 1.032 [0.957 – 1.113] | 0.410 |
| LVIDd | 1.053 [0.991 – 1.118] | 0.095 |
| IVSd | 1.370 [1.177 – 1.596] | <0.001 |
| PWd | 1.271 [1.047 – 1.542] | 0.015 |
| RWT | 3.693 [0.523 – 26.076] | 0.190 |
| LV Mi | 1.027 [1.015 – 1.038] | <0.001 |
| LV ESVi | 1.079 [1.020 – 1.140] | 0.008 |
| LV EDVi | 1.028 [1.003 – 1.054] | 0.028 |
| LVEF | 0.972 [0.924 – 1.021] | 0.259 |
| E | 1.009 [0.994 – 1.024] | 0.244 |
| A | 1.018 [1.007 – 1.029] | 0.001 |
| E/A | 0.492 [0.195 – 1.240] | 0.132 |
| DT | 1.005 [1.001 – 1.009] | 0.010 |
| Mitral lateral s*'* | 0.971 [0.867 – 1.086] | 0.603 |
| Mitral lateral e*'* | 0.881 [0.795 – 0.977] | 0.017 |
| Mitral lateral a*'* | 1.031 [0.947 – 1.123] | 0.480 |
| Mitral medial s*'* | 0.856 [0.720 – 1.017] | 0.076 |
| Mitral medial e' | 0.827 [0.727 – 0.941] | 0.004 |
| Mitral medial a*'* | 0.937 [0.819 – 1.071] | 0.338 |
| E/e*'* average | 1.163 [1.072 – 1.262] | <0.001 |
| LAVi | 1.033 [1.009 – 1.058] | 0.007 |
| RVd | 1.012 [0.962 – 1.066] | 0.640 |
| TAPSE | 1.028 [0.959 – 1.101] | 0.437 |
| RAVi | 1.021 [0.985 – 1.058] | 0.264 |
| LVGLS | 1.051 [0.967 – 1.141] | 0.239 |
| GWE | 0.964 [0.906 – 1.025] | 0.238 |
| GWI | 0.929 [0.875 – 0.986] | 0.015 |
| GCW | 0.943 [0.887 – 1.003] | 0.064 |
| GWW | 1.109 [0.836 – 1.425] | 0.419 |

*A, atrial contraction; a’, peak late (atrial) diastolic annular velocity; BMI, body mass index; CI, confidence interval; DT, deceleration time; E, early diastolic filling; e’, early diastolic annular velocity; EDVi, end diastolic volume index; EF, ejection fraction; ESVi, end-systolic volume index; GCW, global constructive work; GWE, global work efficiency; GWI, global work index; GWW, global wasted work; HR, hazard ratio; IVSd, inter-ventricular septal diameter; LAVi, left atrial volume index; LV, left ventricle; LV GLS, left ventricular global longitudinal strain; LVIDd, left ventricular internal diameter at end-diastole; LV Mi, left ventricular mass index; PWd, posterior wall diameter; PACS, peak atrial contraction strain; PALS, peak atrial longitudinal strain; RAVi, right atrial volume index; RVd, right ventricle diameter; RWT, relative wall thickness; s’, systolic annular velocity; TAPSE, tricuspid annular plane systolic excursion*

Supplementary Table 7. Prognostic assessment of guideline-directed GWI cutoff (1292 mmHg%) using multivariable Cox proportional hazard models

| **Multivariable Cox regressions in different subgroups** | | | | | | | | |
| --- | --- | --- | --- | --- | --- | --- | --- | --- |
|  | **Total Cohort (n=1330)** | | **Normal weight (n=405)** | | **Overweight (n=526)** | | **Obesity (n=399)** | |
|  | **HR [95% CI]** | **p-value** | **HR [95% CI]** | **p-value** | **HR [95% CI]** | **p-value** | **HR [95% CI]** | **p-value** |
| **Female sex** | 0.506 [0.360-0.711] | <0.001 | 0.202 [0.081-0.503] | <0.001 | 0.689 [0.418-1.137] | 0.145 | 0.561 [0.315-0.997] | 0.049 |
| **SBP** | 1.023 [1.014-1.031] | <0.001 | 1.045 [1.027-1.064] | <0.001 | 1.021 [1.009-1.032] | <0.001 | 1.008 [0.990–1.026] | 0.412 |
| **BMI** | 1.027 [0.992-1.063] | 0.130 | 0.853 [0.694-1.050] | 0.133 | 0.925 [0.772-1.108] | 0.398 | 1.043 [0.963-1.129] | 0.303 |
| **GWI < 1292 mmHg%** | 3.358 [2.003-5.628] | <0.001 | 4.891 [1.058-22.605] | 0.042 | 4.182 [1.933-9.045] | <0.001 | 2.476 [1.122-5.461] | 0.025 |

BMI, body mass index; GWI, global work index; SBP, systolic blood pressure
